# Myocardial bridging in patients with hypertrophic cardiomyopathy is not associated with late gadolinium enhancement at cardiac magnetic resonance imaging

**DOI:** 10.1186/1532-429X-15-S1-P158

**Published:** 2013-01-30

**Authors:** Tilman A Hees, Benjamin Herzog, Arnt Kristen, Hugo A Katus, Hassan Abdel-Aty

**Affiliations:** 1 Department of Cardiology, University of Heidelberg, Heidelberg, Germany

## Background

Coronary arteries and their major branches are usually located on the surface of the heart. Infrequently, one portion of coronary arteries is situated intra-myocardially, a condition termed myocardial bridging (MyB). Generally, its prevalence is reported to be around 15% but there is evidence that in patients with hypertrophic cardiomyopathy (HCM) this phenomen is far more frequent with an incidence of up to 41%. Although the effect of MyB is mostly in systoly (not-perfused anyway), its impact on myocardium to induce myocardial fibrosis due to chronic prolonged hypo-perfusion is unknown. Late gadolinium enhancement (LGE) cardiac magnetic resonance (CMR) is able to detect focal myocardial fibrosis. Therefore, we sought to investigate the relationship between MyB and focal LGE often seen in HCM.

## Methods

Methods: 124 patients with HCM (non-obstructive n=87;obstructive n=37;mean-age 61±1 ys, male/female 91/33, NYHA-class 1.9±0.1) were evaluated by CMR and coronary angiography. Both, coronary angiograms and CMR were assessed by two blinded independent observers. Parallel imaging vector-ECG gated 32-channel cine CMR short axis, two and four chamber views were acquired covering the entire left ventricle were acquired using a regular SSFP sequence on a 1.5T Whole Body MRI scanner (Achieva 1.5T, Philips). LGE-CMR (Gadolinium-DTPA:0.2mmol/kg,Magnevist) was performed using similar axes. Areas of fibrosis were identified visually and their anatomic regions were allocated a AHA 17-segment-model. MyB was defined as visual change in luminal compression of an epicardial coronary artery and categorized semi-quantitatively (mild <50%, intermediate 50-75%, severe >75%). Standard nomenclature of coronary artery anatomy was used to define the area affected by MyB.

## Results

MyB was present in 42 of 124 patients (33.9%) with a total number of 82 MyB segments. Most commonly, MyB was present in LAD segments 7 (=22; 26.8%) and 8 (n=20;24.4%). Further MyB segments were LAD 6 (n=7; 8.5%), LAD 9 (n=7;8.5%), LAD 10 (n=5;6.1%), LCX 12 (n=6;7.3%), LCX 14 (n=6; 7.3%), LCX 15 (n=4;4.9%), LCX 13 (n=2;1.2%), LCX 11 (n=1;1.2%), and RCA 4 (n=2; 2.4%). Demographic characteristics did not differ between patients with and without MyB. LGE was observed in 73 patients (58.9%) with a total number of 501 segments. Among the patients with LGE 26 patients (35.6%) had MyB (p=0.62).

## Conclusions

Conclusion: MyB is a common phenomenon in patients with HCM. However, it appears that MyB did not cause myocardial ischemia resulting in ocurrence of LGE.

## Funding

none

